# Polarity Events in the *Drosophila melanogaster* Oocyte

**DOI:** 10.3389/fcell.2022.895876

**Published:** 2022-05-05

**Authors:** Ana Milas, Ivo A. Telley

**Affiliations:** Instituto Gulbenkian de Ciência, Oeiras, Portugal

**Keywords:** par polarity, body axis, mRNA localization, cytoskeletal polarization, follicle cells

## Abstract

Cell polarity is a pre-requirement for many fundamental processes in animal cells, such as asymmetric cell division, axon specification, morphogenesis and epithelial tissue formation. For all these different processes, polarization is established by the same set of proteins, called partitioning defective (Par) proteins. During development in *Drosophila melanogaster*, decision making on the cellular and organism level is achieved with temporally controlled cell polarization events. The initial polarization of Par proteins occurs as early as in the germline cyst, when one of the 16 cells becomes the oocyte. Another marked event occurs when the anterior–posterior axis of the future organism is defined by Par redistribution in the oocyte, requiring external signaling from somatic cells. Here, we review the current literature on cell polarity events that constitute the oogenesis from the stem cell to the mature egg.

## Introduction

Cell polarization is the canonical process of distributing cellular components—from molecules to organelles—unevenly, leading to intracellular and morphological asymmetry. The acquisition and definition of cell functions are often linked to this asymmetry. In broader terms, cell polarization can be viewed as cellular decision-making process generating spatial information. A well-studied example is apicobasal axis formation in epithelial cells. The apical membrane faces the outside of the cell, while the basal membrane contacts the inside. This polarization is the foundation for compartmentalization, organ formation and physical separation of the vertebrate body from the environment (Cereijido et al., 2004; Roignot et al., 2013; Rodriguez-Boulan and Macara, 2014). Another example is the distinction of the differentiating cell from the stemness maintaining cell during asymmetric stem cell division (Knoblich, 2008; Venkei and Yamashita, 2018).

A subclass of metazoans, called bilaterians, define two body axes during embryonic development, the anterior-posterior (AP) axis being the first and the dorsal-ventral (DV) axis following as second symmetry break (Kimelman and Martin, 2012; Anlas and Trivedi, 2021). For most species the AP axis defines head and tail. Some bilaterians, including humans, define a third (left-right) body axis and show, for example, asymmetric organ position (Capdevila et al., 2000). Different animals utilize a range of mechanisms to achieve the symmetry breaks. Vertebrates define these main body axes during embryogenesis (Meinhardt, 2006; Kimelman and Martin, 2012; Bénazéraf and Pourquié, 2013) while invertebrate body axis formation occurs prior or at fertilization (Kimelman and Martin, 2012). Dipterans define the first two body axes prior to fertilization, during late oogenesis.

**FIGURE 1:**
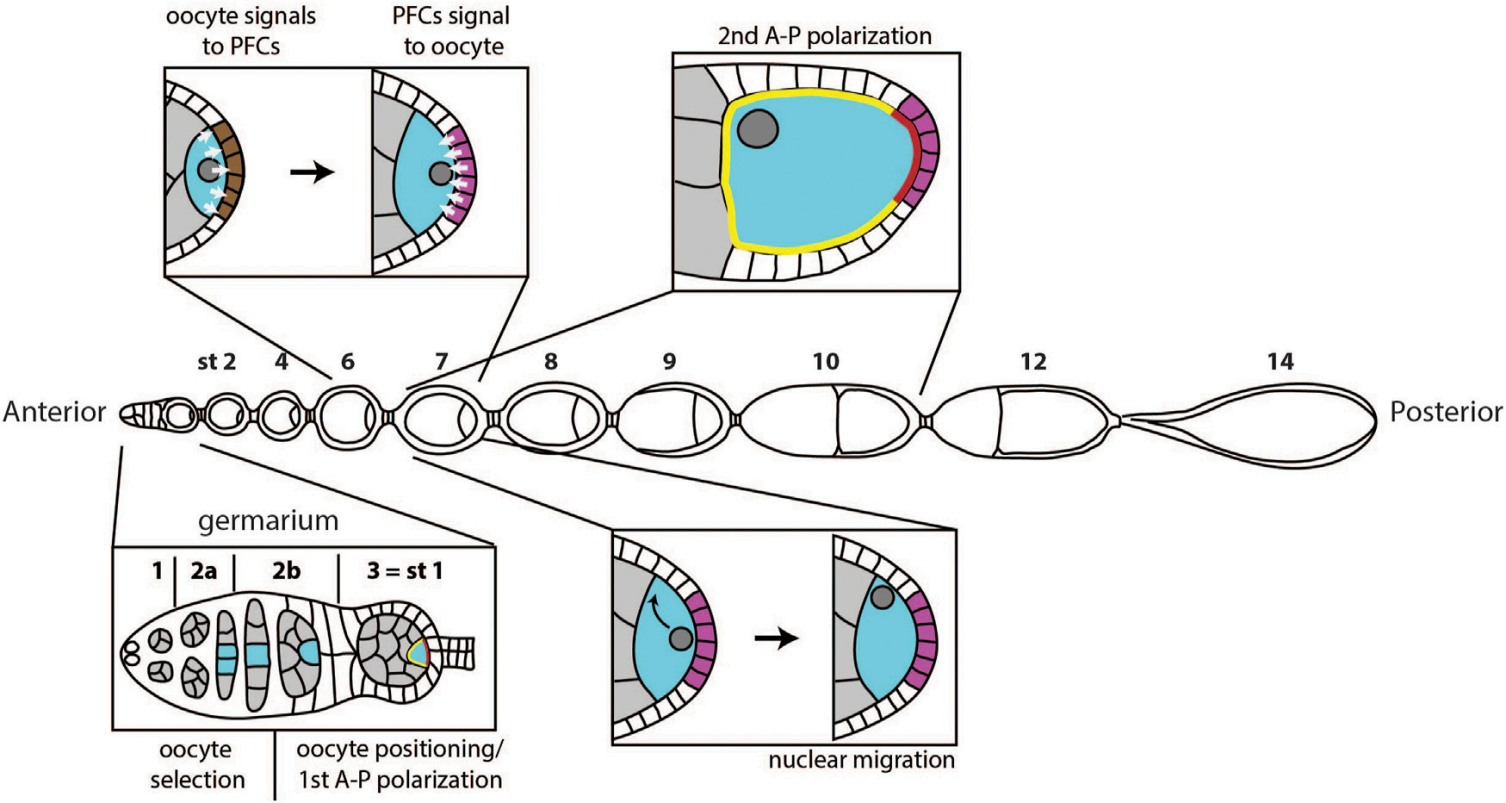
Overview of polarity events during oogenesis. A first polarity event happens in the germarium, when one of the cells in the germline cyst (grey) is selected to become the oocyte (blue). Next, the oocyte moves to the posterior of the germline cyst, and the egg chamber buds from the germarium, marking stage 1 of oogenesis. Around the same time, the oocyte cytoplasm becomes temporarily polarized, with defined anterior (yellow) and posterior (red) domain. At stage 6, the oocyte signals to the follicle cells at the posterior (brown), which causes them to adopt posterior follicle cell fate (magenta). At stage 7, posterior follicle cells (PFCs) signal back to the oocyte. This signal causes migration of the oocyte nucleus to the anterior of the oocyte at stage 7 and triggers a sequence of events that define anterior-posterior polarization of the oocyte between stages 7 and 10.

In the fruit fly *Drosophila melanogaster* oogenesis occurs inside ovarioles, structures composed of the germarium at the anterior tip, and sequentially more mature egg chambers towards the posterior (Figure 1). The germarium hosts germline and somatic stem cells, which divide to give rise to a variety of cell types composing the egg chamber. Germline stem cells derive nurse cells and the oocyte, while follicle stem cells produce somatic follicle cells. Each egg chamber contains one oocyte and 15 nurse cells, surrounded by a layer of follicle cells.

Several polarization events occur during oogenesis for a mature egg to have properly specified axes (Figure 1). If one of these events fails, the system loses one or both axes of asymmetry (Roth and Lynch, 2009). The first symmetry breaking step of oogenesis happens in the germarium, when one of the cells in the germline cyst becomes the oocyte, while others become nurse cells. In the more anterior region of the germarium, follicle cells start surrounding the cyst and play an important role in the subsequent polarization event: the positioning of the oocyte to the posterior of the egg chamber. Positioning of the oocyte is accompanied by budding of the egg chamber from the germarium and the first anterior-posterior polarization of the oocyte. The following two polarization events happen during mid-oogenesis and require communication between the oocyte and follicle cells. First, the oocyte sends a signal to follicle cells at the posterior to induce posterior fate. Next, these cells send a signal back to the oocyte to induce the establishment of the two body axes. Establishment of the anterior-posterior body axis is achieved through polarization of the Par protein network. Par network asymmetry will repolarize the microtubule cytoskeleton, which will enable proper localization of *oskar* and *bicoid* mRNAs. Additionally, the signal from posterior follicle cells leads to the migration of the oocyte nucleus, which defines dorsal-ventral axis of the future embryo.

In this review, we highlight and discuss the current body of knowledge of oocyte polarization in the fruit fly, the molecular players defining events of asymmetry, and we outline the most critical open questions on this topic.

## The First Symmetry Breaking Event and Oocyte Selection

**FIGURE 2:**
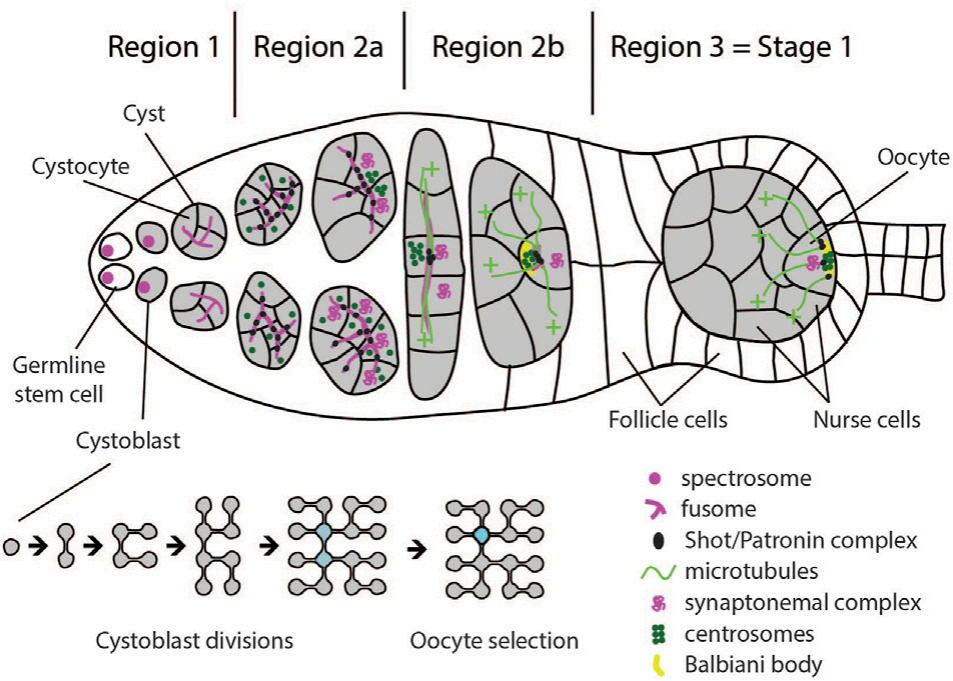
Schematic of a germarium illustrating cellular configurations and polarity events in regions 1–3. Germline stem cells divide asymmetrically and generate a cystoblast. Subsequent divisions occur with incomplete cytokinesis, leaving the cystocytes connected by ring canals. The two cystocytes with most connections become pro-oocytes. Both pro-oocytes, as well as several cells with three ring canals enter meiosis I and accumulate synaptonemal complex in region 2a. By the end of region 2b, only the oocyte remains in meiosis, while all other cells in the cyst start endoreplication and become nurse cells. Germline stem cells and cystoblasts contain spectrosome which develops into the fusome, connecting cells inside the cyst. At region 2a, the Shot/Patronin complex starts accumulating along the fusome, and acts as microtubule-organizing center (MTOC). The cell with most fusome accumulates most Shot/Patronin complex and the largest number of microtubule minus-ends. Oocyte determinants, including centrosomes and mRNAs, are preferentially delivered to this cell, causing it to adopt oocyte fate. In region 2b, oocyte determinants accumulate at the anterior of the oocyte and form the Balbiani body. At stage 3, MTOC and Balbiani body migrate from the anterior to the posterior of the oocyte to define the posterior cortical domain. Around the same time, the oocyte adheres to the follicle cells at the posterior of the germarium, which positions it to the posterior of the germline cyst.

The most anterior tip of the germarium (region 1) hosts a number of germline stem cells (GSC). These cells divide asymmetrically to produce another stem cell and a differentiating daughter cell called cystoblast. This cystoblast undergoes four rounds of incomplete cell divisions giving rise to a cyst of 16 cells, which are called cystocytes. Due to these cell divisions being incomplete, the cystocytes remain connected through cytoplasmic bridges called ring canals. The cystoblast divides into two cystocytes, which go through three more rounds of division and, thus, have four ring canals. Due to the history of divisions, two of the sixteen cystocytes have three, four have two, and eight have only one ring canal (Figure 2, bottom). The two cells with four ring canals are called pro-oocytes, and one of them will differentiate into the oocyte. The other 15 cells in the cyst become nurse cells, which transport mRNAs, proteins, and nutrients to the oocyte through the ring canals (Figure 2) (De Cuevas et al., 1997).

The identity of the oocyte is established following two distinct events. In the nucleus, the oocyte arrests in the prophase of meiosis I, while the other cytocytes exit meiosis I and start the endoreplication cycle. In the cytoplasm, the microtubule network is organised such that, while passing the ring canals, the microtubule minus-ends predominantly accumulate in one of the two pro-oocytes and form a non-centrosomal microtubule organizing centre (ncMTOC) (Theurkauf et al., 1993; Huynh and St Johnston, 2004). This leads to the accumulation of oocyte specific components in this pro-oocyte by means of dynein dependent transport from the other cystocytes (Suter and Steward, 1991; Mach and Lehmann, 1997; McGrail and Hays, 1997; Bolívar et al., 2001; Navarro et al., 2004; Nashchekin et al., 2021). Since all subsequent steps of oocyte polarization can be traced back to cytoskeletal polarization, the selection of one of the two pro-oocytes to become the oocyte is considered the symmetry breaking step of oogenesis (Roth and Lynch, 2009).

It has long been assumed that both pro-oocytes are equally competent to become the oocyte. This assumption was based on the naïve observation that the early transitions of the cell cycle in the two pro-oocytes are indistinguishable. Both pro-oocytes, as well as several cells with three ring canals, enter meiosis I and form synaptonemal complexes between homologous chromosomes (Figure 2). Only as the cyst develops, further progression through meiosis is first restricted to the two pro-oocytes in region 2b, and finally to the oocyte in region 3 of the germarium. All other cells start endocycling, thus becoming nurse cells (Carpenter, 1975; Carpenter, 1994; Röper and Brown, 2004; Nashchekin et al., 2021). However, increasing evidence suggests that the symmetry breaking event of oogenesis occurs already during the first mitotic division of the cystoblast in region 1 of the germarium. The evidence supporting this notion comes from the analysis of assembly and distribution of the “fusome” (de Cuevas and Spradling 1998). The fusome is a germline specific, membranous, branched intracellular structure that runs through the ring canals and connects all cystocytes (Lin et al., 1994). The cystoblast inherits the fusome from the germline stem cell. The inherited fusome then serves to orient the cell division of the cystoblast by anchoring one pole of the spindle. After division, only one cell inherits the fusome while the other cell initially lacks it. During interphase, a new fusome forms in the ring canal. The fusomes from both daughter cells migrate to each other and fuse, which ultimately results in an asymmetric distribution with one cell having the original fusome inherited from the germline stem cell plus half of the newly formed fusome, while the other cell has only half of the newly formed fusome. This asymmetric distribution continues to occur during the next three divisions. As a result, the cell that inherited the original fusome in the first division will end up having the largest amount of fusome (Lin and Spradling, 1995; Deng and Lin, 1997; De Cuevas and Spradling, 1998; Villa-Fombuena et al., 2021).

Following the observation that microtubules form along the fusome, the hypothesis was put forward that polarization of the microtubule network is a direct consequence of fusome polarity (Grieder et al., 2000). In agreement with this idea was the observation that the *Drosophila* homologue of Spectraplakin, Short Stop (Shot), a component of the fusome, is necessary for oocyte specification, and appears to stabilize microtubules and link them to the fusome (Röper and Brown, 2004). Recent data further supported this model by showing that Shot stabilizes microtubules by recruiting microtubule minus end stabilizing protein Patronin to the fusome, and that Patronin is necessary for oocyte specification (Nashchekin et al., 2021). Patronin stabilizes microtubule minus-ends in the cell with the largest portion of fusome, thereby establishing a weakly polarized microtubule network. This initial asymmetry is reinforced through positive feedback since dynein transports Patronin bound microtubules from neighboring cells to the cell that already contains most Patronin. Finally, a now highly polarized microtubule network is utilized by dynein to transport oocyte determinants into this cell (Figure 2) (Nashchekin et al., 2021).

If this model of oocyte specification is correct, then the cell that inherits the original fusome in the cystoblast division also accumulates most of the expressed Patronin and eventually becomes the oocyte. Indeed, two studies showed that centrosomes, *oskar* and *orb* mRNAs preferentially accumulate in the cell with the most fusome (Grieder et al., 2000; Cox and Spradling, 2003).

## Positioning of the Oocyte to the Posterior of the Egg Chamber

As the cyst moves through region 2 of the germarium, it is surrounded by a layer of somatic follicle cells. These cells arise from asymmetric divisions of follicle stem cells that reside in the middle of the germarium. Follicle cells further differentiate into either main body follicle cells, or the precursors of stalk or polar cells (reviewed in Rust and Nystul, 2020). Oocyte determination and initial steps of follicle cell differentiation seem to be independent. However, many subsequent steps of polarization of both the oocyte and the layer of follicle cells depend on their mutual communication (see Merkle et al., 2020 for a recent review on signaling between soma and germline throughout oogenesis).

**FIGURE 3:**
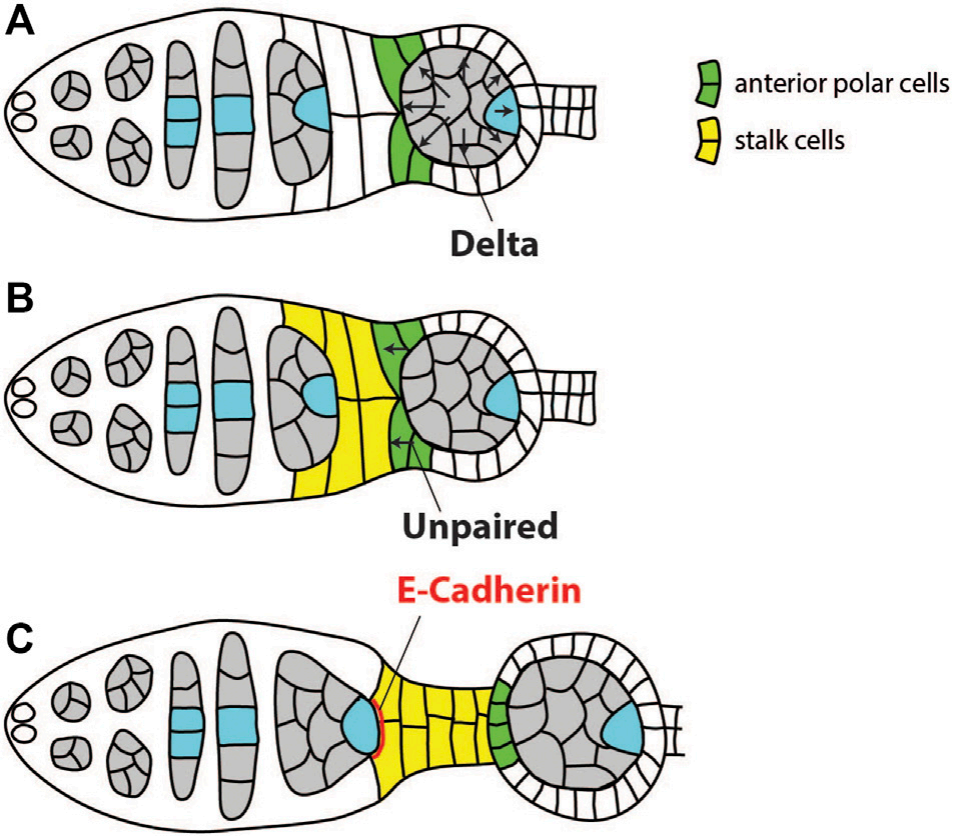
Schematics of signaling events and cell-cell interactions required for positioning of the oocyte to the posterior of the egg chamber. **(A)** Germline cyst in region 3 expresses Notch ligand Delta to induce differentiation of the anterior polar cells (green). **(B)** The polar cells express Unpaired, leading to differentiation of stalk cells (yellow). **(C)** Both follicle cells and the oocyte upregulate DE-cadherin to increase mutual adhesion and position the oocyte to the posterior of the egg chamber. Adapted from Torres et al. (2003).

The first round of signaling from the germline cyst to the soma activates Notch and JAK/STAT pathways to induce differentiation of polar and stalk follicle cells (Figure 3). These cells are in turn required to position the oocyte to the posterior of the egg chamber. Before this round of signaling, undifferentiated precursors of stalk/polar follicle cells separate the younger cyst in region 2b from the older cyst in region 3 (Tworoger et al., 1999). The older cyst expresses Notch ligand Delta, which activates Notch in the surrounding follicle cells. This causes precursors of stalk/polar cells that are in direct contact with the anterior of the older cyst to differentiate into polar cells (Figure 3A) (Grammont and Irvine, 2001; López-Schier and St Johnston, 2001). These polar cells will then express Unpaired which will activate JAK-STAT signaling in more anterior stalk/polar precursors, leading to their differentiation into stalk cells (Figure 3B) (McGregor et al., 2002; Torres et al., 2003). Unpaired is unable to activate JAK-STAT in cells which previously underwent high activation of Notch. Thus, it will not act on polar cells themselves, or on follicle cells surrounding older cyst (Assa-Kunik et al., 2007). Newly differentiated stalk cells form a stalk which directly contacts the younger germline cyst in region 2b. Both stalk cells and the oocyte express high levels of DE-cadherin, which causes the oocyte to adhere to follicle cells at the posterior, thereby positioning the oocyte to the posterior of the egg chamber (Figure 3C) (Godt and Tepass, 1998; González-Reyes and St Johnston, 1998a). Thus, in this round of signaling information is transferred from the older to the younger cyst through a relay mechanism to correctly differentiate polar and stalk cells, and to position the oocyte at the posterior of the egg chamber (Torres et al., 2003). It is not known how the oocyte in the first cyst, which does not have a leading older cyst, is positioned to the posterior. Stalk cells will also contribute to the establishment of the polar cells at the posterior pole of the oocyte (Torres et al., 2003; Assa-Kunik et al., 2007). These posterior polar cells, as well as the correct positioning of the oocyte, will be crucial in later stages of oogenesis when the new round of signaling between the oocyte and posterior follicle cells (PFCs) takes place to establish both AP and DV axis (González-Reyes and St Johnston, 1994; Grammont and Irvine, 2002).

## First Round of Oocyte Anterior-Posterior Polarization

**FIGURE 4:**
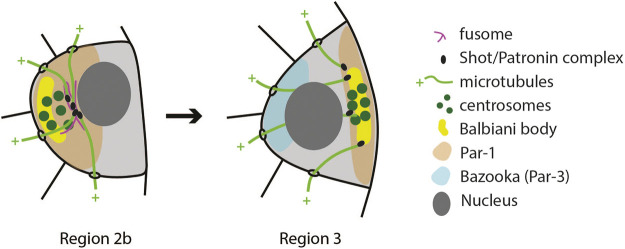
Schematics of the first polarity event in the oocyte. The microtubule cytoskeleton reorganizes in the transition from Region 2b to Region 3 so that their nucleation sites (Shot/Patronin) are now at the posterior end. This causes the Balbiani body to reposition from the anterior to the posterior. Similarly, Par-1 localization changes from anterior to posterior, while Bazooka (Par-3) shows antagonistic localization in Region 3 (Vaccari and Ephrussi, 2002).

Positioning of the oocyte to the posterior of the egg chamber is accompanied by changes in the oocyte cytoplasm. Components that were transported to the oocyte during the selection phase, such as centrosomes, Orb, BicD and Egl protein, *oskar* and *orb* mRNAs, are initially located at the anterior of the oocyte and form a structure called Balbiani body (Cox and Spradling, 2003). Minus–ends of microtubules, which facilitated the transport of Balbiani body components to the oocyte, are also accumulated at the anterior. When the oocyte moves through region 3, the microtubule network is reorganized so that minus–ends are more frequently found at the posterior (Grieder et al., 2000). This is followed by relocalisation of Balbiani body components to the posterior of the oocyte where they form a tight crescent to define the posterior oocyte cortex (Figure 4). If this step fails, the oocyte loses its fate and becomes a nurse cell by exiting meiosis and becoming polyploid (Huynh et al., 2001a). Mechanisms involved in early oocyte polarization are not well understood. However, a collection of experimental evidence suggests that it depends on all *Drosophila* homologues of *par* genes, as well as polarity proteins aPKC and Cdc42. When any of these genes are lacking, the oocyte de-differentiates into a nurse cell (Cox et al., 2001a; Cox et al., 2001b; Huynh et al., 2001a; Huynh et al., 2001b; Benton et al., 2002; Vaccari and Ephrussi, 2002; Martin and St Johnston, 2003; Leibfried et al., 2013). Par proteins are a highly conserved group of polarity proteins originally identified in *Caenorhabditis elegans*. In the *C. elegans* zygote, Par-1 and Par-2 localize to the posterior membrane, while Par-3 and Par-6 form a complex with aPKC and localize to the anterior. Polarity is maintained through mutual phosphorylation of Par proteins. Par-1 excludes Par-3 from the posterior, while aPKC excludes Par-1 from the anterior. Another highly conserved polarity protein, small GTPase Cdc-42, is also required at the anterior where it activates aPKC (reviewed in Lang and Munro, 2017; St Johnston, 2018).

Initial efforts to determine the localization of Par proteins during early oogenesis showed that Bazooka (the *Drosophila* homologue of Par-3) and aPKC localize to adherens junctions that form around the ring canals (Cox et al., 2001b), while Par-1 localizes to the fusome (Cox et al., 2001a; Huynh et al., 2001a; Shulman et al., 2000). This work also suggested that Bazooka, aPKC and Par-1 do not depend on each other for their localization (Cox et al., 2001b; Huynh et al., 2001b). However, using isoform specific antibodies, Vaccari and Ephrussi (2002) detected Bazooka at the anterior, and Par-1 at the posterior pole of the oocyte (Figure 4). They also showed that Bazooka extends to the posterior in *par-1* mutants, while Par-1 remains anterior in *baz* mutants. This suggests that Par polarity in the early oocyte could be maintained by mutual antagonism between the Baz/Par6/aPKC complex and Par-1. Cdc42 localizes to the anterior of early oocytes, and in *cdc42* mutant egg chambers Bazooka localization is lost. Inversely, anterior localization of Cdc42 is lost in *baz* and *apkc* mutants (Leibfried et al., 2013).

It is unclear what triggers the polarization of the Par network in early oogenesis. But, since this event coincides with cadherin-mediated interactions between follicle cells and the oocyte, it has been suggested that a signal from follicle cells might play a role (Huynh and St Johnston, 2004; Roth and Lynch, 2009). This view has been supported by the finding that extracellular matrix receptor dystroglycan is required in the germline to polarize the oocyte at this stage (Deng et al., 2003). In addition, signaling between follicle cells and the oocyte is required for polarization of the Par network in later stages of oogenesis (Doerflinger et al., 2006).

At this stage of oogenesis, a number of open questions are eminent. It is not clear how *par* genes maintain oocyte fate, or how they are involved in relocalisation of the Balbiani body. It is also not clear if the Par network needs to be polarized at this stage to maintain oocyte fate. The microtubule network is likely a downstream target of Par polarity. This is based on the finding that microtubule minus–ends do not relocalise to the posterior cortex in any of the *par* mutants (Cox et al., 2001b; Huynh et al., 2001a; Benton et al., 2002; Vaccari and Ephrussi, 2002; Martin and St Johnston, 2003; Leibfried et al., 2013). However, Par-1 was later shown to inhibit microtubule nucleation at the posterior in later stages of oogenesis (Parton et al., 2011; Nashchekin et al., 2016). Therefore, it is not clear how Par-1 localization at the posterior could induce localization of minus ends at the posterior in earlier stages. In addition, posterior localization of Par-1 at this stage requires an intact microtubule network, suggesting that microtubule polarity and Par protein localization are interdependent (Vaccari and Ephrussi, 2002).

Another possible target of the Par network is the actin cytoskeleton; *cdc42, apkc* and *baz* mutants show disrupted actin cytoskeleton, while treatment with Latrunculin A abolishes translocation of Orb from anterior to posterior of the oocyte (Leibfried et al., 2013). Additionally, components of the dynein/dynactin complex could also be targets of Par proteins (Huynh and St Johnston, 2004). This idea grew from the observation that translocation of proteins and centrosomes does not occur correctly in *Dhc*, *BicD* or *egl* mutants (Huynh and St Johnston, 2000; Bolívar et al., 2001; Vaccari and Ephrussi, 2002).

## Signal From the Oocyte Specifies Posterior Follicle Cells

**FIGURE 5:**
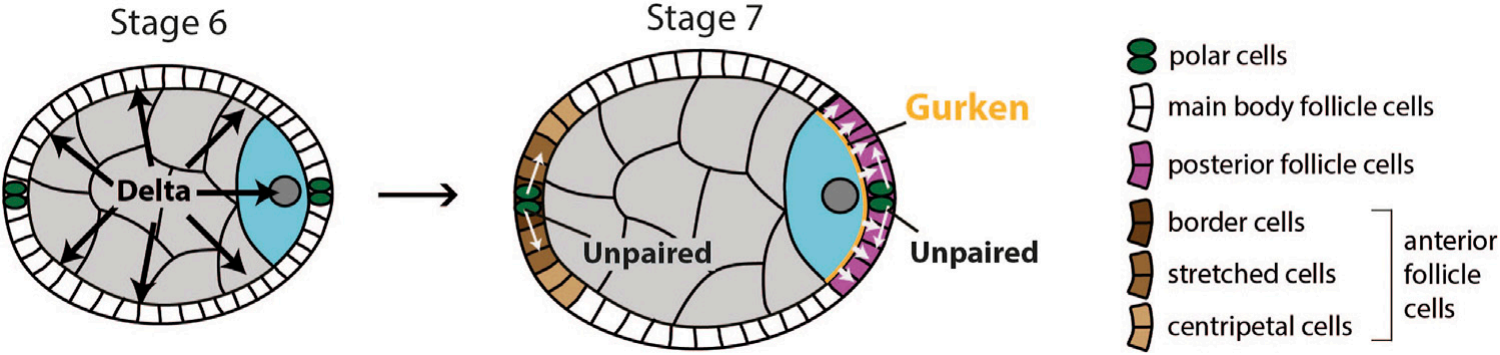
Schematics of signaling events required for differentiation of anterior and posterior follicle cells. At stage 6, the germline expresses Delta, which causes the surrounding layer of follicle cells to become competent to respond to JAK-STAT signaling. At stage 7, polar cells secrete Unpaired to activate JAK-STAT pathway in surrounding follicle cells. At the anterior, Unpaired acts as morphogen to specify three types of anterior follicle cells. At the posterior, the oocyte secretes Gurken to activate EGF pathway in adjacent follicle cells. Activation of both JAK-STAT and EGF pathway causes these follicle cells to adopt posterior fate.

A second round of signaling between the oocyte and follicle cells involves Notch, JAK-STAT and EGF pathways, and occurs during stages 6 and 7 of oogenesis. At this point, Delta is once again upregulated in the germline and activates Notch signaling in the surrounding follicle cells (López-Schier and St Johnston, 2001). The activation of Notch signaling has two main effects on follicle cells. First, it initiates a switch from the mitotic cycle to the endoreplication cycle, which inhibits the proliferation of follicle cells (Deng et al., 2001; López-Schier and St Johnston, 2001). Second, it provides competency to respond to JAK-STAT signaling, which is activated by secretion of Unpaired from polar cells at the anterior and posterior ends of the egg chamber (Figure 5). This leads to specification of terminal cell fate in surrounding epithelial cells at both the anterior and the posterior end of the egg chamber. At the anterior, Unpaired functions as a morphogen to specify three types of anterior follicle cells as a function of distance from the polar cells: 1) border cells, 2) stretched cells, and 3) centripetal cells. Border cells receive the highest, and centripetal cells the lowest levels of Unpaired (Xi et al., 2003). Proper differentiation of anterior follicle cells is not necessary for establishment of the anterior-posterior (AP) axis but is important for other aspects of egg chamber development (Wu et al., 2008).

For posterior follicle cells to differentiate correctly, the EGF signaling pathway needs to be activated. If that does not happen, follicle cells at the posterior pole of the egg chamber will differentiate into the three types of anterior follicle cells mentioned earlier (González-Reyes et al., 1995; Roth et al., 1995; González-Reyes and St Johnston, 1998b). EGFR in the follicle cells is activated by TGFɑ-like ligand Gurken (Neuman-Silberberg and Schüpbach, 1993). Gurken is secreted by the adjacent oocyte and activates EGFR in around 200 follicle cells (Neuman-Silberberg and Schüpbach, 1993; González-Reyes and St Johnston, 1998b). This leads to transcription of several target genes of EGF signaling such as *pointed* (Morimoto et al., 1996), *midline* and *H15* (Fregoso Lomas et al., 2013)*,* determinants of posterior follicle cell fate. Importantly, Gurken can activate EGFR only in follicle cells that have previously received Notch and JAK-STAT signaling (Xi et al., 2003). Thus, all three pathways are needed for posterior follicle cells (PFCs) to correctly differentiate and to signal back to the oocyte, which establishes the AP and DV body axes.

## Posterior Follicle Cells Signal Back to the Oocyte

The role of the follicle cells in establishing anterior-posterior polarity of the oocyte has first been proposed in the early 1990s, based on the finding that Notch and Delta genes are required in follicle cells for proper localization of *bicoid* mRNA in the oocyte (Ruohola et al., 1991; Ruohola-Baker et al., 1994). González-Reyes and St Johnston (1994) showed that in a mutant in which the oocyte is mispositioned to the centre of the egg chamber, posterior follicle cells adopt anterior fate. This led them to propose a model according to which the oocyte signals to the follicle cells at the posterior to induce posterior fate, which in turn signal back to the oocyte to promote reorganization of the microtubule cytoskeleton. Soon after this finding, it was determined that the signal coming from the oocyte is Gurken, which activates the EGF signaling pathway in the PFCs. This work also confirmed the need for a signal coming from the posterior follicle cells, by showing that components of the EGFR network in follicle cells were necessary for proper localization of *oskar* and *bicoid* mRNA, organization of the microtubule cytoskeleton, and positioning of the nucleus (González-Reyes et al., 1995; Roth et al., 1995). However, more than 25 years after the signal coming from the oocyte has been elucidated, the molecular nature of the returning signal from the follicle cells that polarizes the oocyte remains unknown.

In order to identify the returning signal, several forward genetic screens for downstream targets of Notch, JAK/STAT and EGFR in follicle cells have been performed. However, they have not been successful in identifying the gene that encodes the signal molecule (Pai et al., 2000; Chen and Schüpbach, 2006; Sun et al., 2011; Wittes and Schüpbach, 2019). Evidence coming from mosaic analysis of mutants in EGF and JAK-STAT signaling components suggests that the signal is transmitted in a local fashion. These studies looked at localization of *oskar* mRNA and Staufen protein [an RNA-binding protein that can be used as a proxy for *oskar* mRNA localization (St Johnston et al., 1991)] in egg chambers in which mutant cell clones encompass only a subset of the PFCs. Both *oskar* mRNA and Staufen protein localize normally at the regions of the oocyte cortex that face wildtype PFCs, while mislocalisation is observed only in regions facing mutant follicle cells (Frydman and Spradling, 2001; Xi et al., 2003).

It is also unknown what the immediate target of the PFC signal is once it reaches the oocyte. The first sign of AP polarity identified to date is activation of non-muscle Myosin II at the posterior of the oocyte, and this does not happen in *grk* mutants, in which PFCs do not differentiate correctly (Doerflinger et al., 2022). However, it is unclear if this change is the direct target of the signal. On the other hand, the oocyte nucleus has to migrate from the posterior to the anterior of the oocyte to define the dorsal side of the egg chamber. In *grk* mutants, the oocyte is not released from the posterior anchor and cannot migrate (Zhao et al., 2012). All the evidence suggests that a PFC signal is necessary to establish both the AP and DV axis of the oocyte. However, the establishment of the two axes seems to be independent since the nucleus migrates normally in *par-1* mutants, which do not properly establish the AP axis (Zhao et al., 2012). This also raises the possibility that the PFCs send two different signals to polarize the two main body axes.

## Migration of the Oocyte Nucleus and Dorsal-Ventral Axis Formation

One of the downstream targets of the unidentified PFC signal is the movement of the nucleus from the posterior pole to the anterior at stage 7. Once it reaches the anterior, the nucleus is anchored to the oocyte membrane in contact with follicle cells. When the migration is completed, the nucleus accumulates high levels of *grk* mRNA and protein, and one more round of EGF signaling follows inducing dorsal fate in adjacent follicle cells (Neuman-Silberberg and Schüpbach, 1993; Roth et al., 1995; Schüpbach, 1987, see Merkle et al., 2020 for recent review on downstream targets of EGFR activation in dorsal follicle cells).

In the mutants producing bi-nucleated oocytes due to a defect in cystoblast cytokinesis, both nuclei move to the anterior and induce dorsal fate in adjacent follicle cells. Additionally, both nuclei choose the position randomly with regard to each other (Roth et al., 1999). Thus, the nucleus can be localized at any position of the oocyte anterior margin, meaning that, prior to nuclear movement, the oocyte lacks any dorsal-ventral asymmetry. This attributes the migration of the nucleus to the specific point of the margin a symmetry breaking event.

The movement of the nucleus across the oocyte has been well characterized using live imaging (Zhao et al., 2012; Tissot et al., 2017, reviewed in; Bernard et al., 2018). Studies have shown that microtubules nucleating at the posterior pole of the oocyte push the nucleus to the anterior. First, Zhao et al. (2012) showed that centrosomes, which are transported to the posterior at stage 1 of oogenesis, are the main nucleators of microtubules. However, detailed 3D analysis of migratory paths revealed that there are complementary mechanisms driving nuclear movement. While centrosomes control one migratory path, microtubule-associated protein Mud/NuMA, promotes a separate route (Tissot et al., 2017). This mechanistic redundancy provides robustness to the process of nuclear migration. In addition, it explains why centrosomes are not necessary for the correct positioning of the nucleus (Stevens et al., 2007). In the absence of centrosomes, nucleus movement is mediated either by the Mud/NuMA pathway (Tissot et al., 2017), or by acentrosomal microtubule organizing centers that form behind the nucleus and provide the pushing force for nuclear migration (Zhao et al., 2012).

Although migration of the nucleus has been well described, it is not clear how the PFC signal triggers the movement of the nucleus. It has been suggested that the signal releases the nucleus from the posterior anchor (Zhao et al., 2012). This is based on the observation that active centrosomes are localized behind the nucleus already at the stage 5 of oogenesis. These centrosomes nucleate microtubules, inducing indentation of the nucleus at the posterior. However, the nucleus is set in motion only following the PFC signal at stage 7. In *grk* mutants, the nucleus still maintains posterior indentation, but fails to migrate since the pushing force remains countered by an anchor that keeps the nucleus in place (Zhao et al., 2012). Once the nucleus has reached its final position, it needs to be anchored (Guichet et al., 2001). If anchoring is omitted, the nucleus is found in the middle of the oocyte, which has been referred to as a floating phenotype (Bernard et al., 2018). Not much is known about the mechanisms of the nucleus anchoring. However, microtubules play a role since a floating phenotype is observed when microtubules are depolymerized after the migration is completed (Januschke et al., 2006).

## Second Round of Oocyte Anterior-Posterior Polarization

**FIGURE 6:**
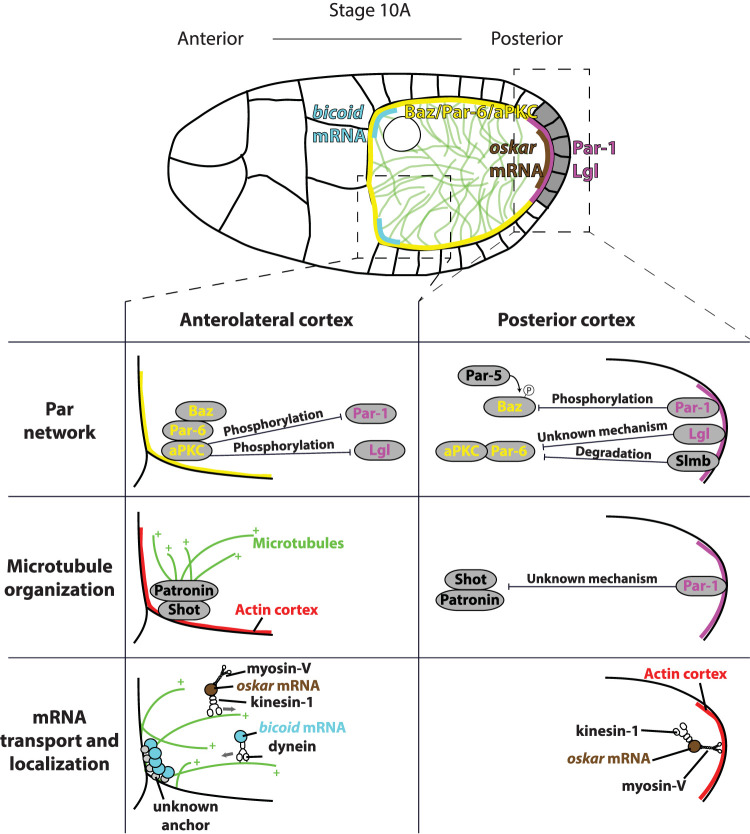
Top: overview of anterior-posterior polarization of the oocyte in mid-oogenesis. Signal from PFCs (grey) induces establishment of posterior (magenta) and anterolateral (yellow) Par domains. Par polarity induces polarization of microtubule cytoskeleton (green) by inhibiting microtubule nucleation at the posterior. Polarized microtubule cytoskeleton directs delivery of *bicoid* mRNAs (blue) to the anterior and *oskar* mRNA (brown) to the posterior. Bottom: Detailed schematics showing three processes necessary for the oocyte polarization. Par network polarity: at the anterior, Bazooka/Par-6/aPKC complex binds to the membrane through interaction between Bazooka and membrane lipids. aPKC phosphorylates both Par-1 and Lgl to exclude them from the anterolateral membrane. At the posterior, Par-1 phosphorylates Bazooka, which is excluded from the posterior membrane following the binding by Par-5. In addition, Lgl and Slmb exclude aPKC/Par-6 complex from the posterior through not well defined mechanisms. Microtubule organization: at the anterior, Shot binds to actin and recruits Patronin. Patronin binds minus ends of existing microtubules, which template growth of new microtubules. At the posterior, Par-1 excludes Shot/Patronin through unknown mechanism to inhibit posterior nucleation of microtubules. mRNA transport and localization: at the anterior, dynein delivers *bicoid* mRNA to the cortex by moving towards the minus ends of the microtubules. *bicoid* is anchored only at the anterior of the oocyte by an unidentified linker. Kinesin-1 removes *oskar* mRNA from the anterolateral cortex by moving towards the plus ends of the microtubules. At the posterior, myosin-V anchors *oskar* mRNA to the cortical actin at the posterior of the oocyte, where the microtubule nucleation is inhibited.

The final goal of establishing anterior-posterior polarity of the oocyte is the robust and precise delivery of anterior and posterior determinants, which will specify the poles of the future embryo. The anterior region will develop into the head while the posterior region will become the abdomen of the fly. The main posterior determinant is *oskar* mRNA (Lehmann and Nüsslein-Volhard, 1986), and the anterior determinant is *bicoid* mRNA (Frohnhöfer and Nüsslein-Volhard, 1986). The process of anterior-posterior polarization of the oocyte occurs from stage 7 to stage 10 and can be divided into three steps (Figure 6). First, an asymmetry of the Par network is established, with Par-1 at the posterior and Bazooka/Par6/aPKC complex at the anterior cortex. Next, Par-1 inhibits microtubule nucleation at the posterior, which polarizes the microtubule cytoskeleton. Finally, motor protein-based transport of mRNAs on a polarized microtubule network leads to asymmetric mRNA localization.

### Polarization of the Par Protein Network

The first sign of Par polarity is the appearance of a Par-1 crescent at the posterior of the oocyte at stage 7 of oogenesis (Shulman et al., 2000; Tomancak et al., 2000). Interestingly, at this stage Bazooka and Par-6 are also detected at the posterior, where they colocalize with Par-1. During stages 8 and 9, the Par-1 crescent intensifies and expands, while Bazooka and Par-6 gradually disappear from the posterior, and re-localise to the anterolateral cortex during stage 9 (Doerflinger et al., 2010; Jouette et al., 2019). Two other Par proteins, Par-4/LKB1 and Par-5/14-3-3, do not show any polarized distribution. Par-4 is uniformly distributed around the cortex (Martin and St Johnston, 2003), while Par-5 is detected in the cytoplasm (Benton et al., 2002). In addition to Par proteins, tumour suppressor Lethal (2) giant larvae (Lgl) is required for the polarization of the oocyte. Lgl also shows a polarized distribution and localizes to the posterior of the oocyte (Figure 6) (Tian and Deng, 2008; Doerflinger et al., 2010).

According to the current model, the Par polarity in the oocyte is maintained by mutual phosphorylation of posterior and anterior Par proteins. At the posterior, the main kinase is Par-1, which phosphorylates Bazooka to reduce its affinity for, and to exclude it from, the membrane. Since Bazooka is a scaffold protein that recruits Par-6 and aPKC to the membrane, exclusion of Bazooka also removes Par-6 and aPKC from the posterior. Additionally, Lgl binds to aPKC/Par-6 complex and inhibits aPKC activity. At the anterolateral cortex, aPKC phosphorylates both Par-1 and Lgl to exclude them from this domain (St Johnston and Ahringer, 2010). However, while evidence for some parts of this mechanism is strong, some have been inferred from other organisms and lack strong support in experiments using the *Drosophila* oocyte.

Evidence that Par-1 is required for Bazooka exclusion from the posterior is convincing. Phosphorylation of Bazooka by Par-1 has been shown both *in vitro*, by using biochemical assays; as well as *in vivo*, in both epithelia and the oocyte. Par-1 phosphorylates Bazooka on two conserved serines to generate a 14-3-3 binding site. Binding of 14-3-3/Par-5 to Bazooka disrupts its oligomerization and interaction with aPKC (Benton and St Johnston, 2003). In addition, in *par-1* mutants Bazooka is localized all around the oocyte cortex; and a non-phosphorylatable form of Bazooka also shows uniform localization (Benton and St Johnston, 2003; Doerflinger et al., 2010). However, while phosphorylation of Bazooka by Par-1 is essential for its exclusion, it might not be sufficient. At stages 7 and 8, Par-1 and Bazooka co-localize at the posterior, and Bazooka exclusion is only observed at late stage 9 (Doerflinger et al., 2010; Jouette et al., 2019). Recent work has suggested the role of membrane trafficking and dynein-mediated transport in maintenance of Bazooka asymmetry (Jouette et al., 2019).

While the requirement of Par-1 for Bazooka localization is clear, contradicting observations concerning how Bazooka influences Par-1 cortical recruitment have been made. One study reported a *baz* mutant in which Par-1 cortical localization is lost (Becalska and Gavis, 2010). Another study reported that Par-1 localizes all around the cortex in *baz* mutant (Doerflinger et al., 2010). When the conserved aPKC phosphorylation site is mutated, Par-1 localizes all around the cortex (Doerflinger et al., 2006, Doerflinger et al., 2010). Additionally, aPKC, as well as its binding partner Par-6, is excluded from posterior in mid-oogenesis (Doerflinger et al., 2010; Morais-de-Sá et al., 2014). From this, it has been inferred that aPKC phosphorylates Par-1 to exclude it from the anterolateral cortex (Doerflinger et al., 2010). However, direct evidence for the requirement of aPKC and Par-6 in oocyte polarization is still missing. The reasoning behind is that most of the oocytes which are mutant for *apkc* and *par-6* lose their fate and revert to that of nurse cells. This problem has been partially circumvented by studying escapers—egg chambers that overcome the early defects and develop to stage 9–10. By analysing escapers of a strong *apkc* mutant, Tian and Deng (2008) found mislocalization of Staufen in half of the oocytes. Conversely, Doerflinger et al. (2006) reported that these escapers develop normal anterior-posterior axis at stage 9. Similarly, hypomorphic aPKC alleles, which lack either kinase activity or Par-6 binding site, do not cause polarity defects (Kim et al., 2009). Additionally, Huynh et al. (2001b) reported that *par-6* escapers produce normal eggs. More recently, it has been reported that Slmb, the substrate specificity subunit of the SCF E3 ubiquitin ligase, excludes Par-6 and aPKC from the posterior by targeting them for degradation (Morais-de-Sá et al., 2014).

### Polarization of Microtubule Cytoskeleton

While exact mechanisms of Par asymmetry establishment in mid-oogenesis remain unclear, much more is known about the downstream polarity event: the organization of the microtubule cytoskeleton. Initially, microtubule organization in the oocyte was inferred from final distributions of cargoes and motor proteins. This has led to a view that microtubules are highly polarized along the anterior-posterior axis, with minus ends at the anterior, and plus ends at the posterior (Clark et al., 1994; Clark et al., 1997). Immunostaining of microtubules has led to the model of microtubules being nucleated at the anterior and lateral cortex of the oocyte, but not at the posterior (Theurkauf et al., 1992; Cha et al., 2002; Serbus et al., 2005), while Januschke et al. (2006) proposed that nucleation occurs predominantly from the oocyte nucleus.

Our current understanding of microtubule organization greatly benefited from the use of high-resolution live imaging. Visualization of *oskar* mRNA particles showed that they are transported in all directions, with only a small bias towards the posterior (Zimyanin et al., 2008). This suggested that the microtubule network is only marginally polarized. Parton et al. used live imaging of microtubule plus end marker EB1-GFP to show that the microtubule network is highly dynamic, and only subtly polarized; around 60% of the microtubules grow towards the posterior, and 40% towards the anterior (Parton et al., 2011). At the anterolateral cortex of the oocyte, microtubules are organized by non-centrosomal microtubule organizing centers (ncMTOCs). These ncMTOCs are composed of spectraplakin, Shot and a microtubule minus end–binding protein, Patronin. Shot binds to the cortical f-actin, and recruits Patronin to form cortical ncMTOC (Figure 6). The Shot/Patronin complex does not nucleate microtubules but captures existing microtubule minus ends, which template growth of new microtubules (Nashchekin et al., 2016). A line of studies showed that Par-1 is a major effector of microtubule organization in the oocyte. In *par-1* mutants, the exclusion of Shot and Patronin from the posterior cortex is lost (Nashchekin et al., 2016), as is the suppression of microtubule nucleation at the posterior (Shulman et al., 2000; Parton et al., 2011). In contrast, expression of the non-phosphorylatable form of Par-1, which localizes all around the cortex, causes loss of all cortex-associated microtubules (Doerflinger et al., 2010). Whether Par-1 interacts directly with the Shot/Patronin complex to exclude it from the membrane, or whether it blocks its cortical recruitment through indirect mechanism, remains to be determined.

### Localization of mRNAs

The final step of anterior-posterior polarization of the oocyte is the delivery of *oskar* mRNA to the posterior, and of *bicoid* mRNA to the anterior of the oocyte. It has been known for a long time that the localization of both mRNAs depends on microtubules (Pokrywka and Stephenson, 1991; Clark et al., 1994). These early results led to the model of mRNAs being transported towards the opposite poles by motor proteins moving in opposite directions on a highly polarized microtubule cytoskeleton (Figure 6, bottom). This idea was corroborated with the finding that correct *oskar* mRNA localization requires plus-end directed motor protein kinesin-1 (Brendza et al., 2000), while localization of *bicoid* mRNA is affected when the Dynein/Dynactin complex is disrupted (Duncan and Warrior, 2002; Januschke et al., 2002). However, as it became clear that the microtubule cytoskeleton is not as polarized as was initially assumed, novel mechanisms of mRNA transport and localization have been considered.

Once again, live imaging was essential for our current understanding of this process. As mentioned previously, high-resolution time-lapse imaging and image analysis revealed that *oskar* mRNA localizes by biased random walk along a weakly polarized microtubule cytoskeleton (Zimyanin et al., 2008). Similarly, Trovisco et al. (2016) found that *bicoid* is randomly transported by dynein walking toward the minus-ends of the microtubules. Computer simulations suggested that the existence of an anterior-posterior gradient of cortical microtubule nucleation is already sufficient to account for the localization of mRNAs (Khuc Trong et al., 2015). However, recent experiments suggested that dynactin is necessary to protect growing plus–ends of MTs from depolymerization, making them long enough for *oskar* to be delivered to the posterior (Nieuwburg et al., 2017). In addition, both *bicoid* and *oskar* mRNAs seem to require anchoring for stable localization. FRAP and photo-conversion experiments of fluorescently labelled *bicoid* mRNA showed slow turnover kinetics of these particles suggesting that they are stably anchored (Trovisco et al., 2016). These observations led the authors to propose that dynein delivers *bicoid* mRNA to a broader anterolateral region by walking towards the minus end of the microtubules. *bicoid* is anchored by an unknown mechanism, which is active only at the anterior, but not at the lateral cortex. The molecular nature of this mechanism is unknown, but it is independent of microtubule dynamics and polarization (Trovisco et al., 2016). Interestingly, *bicoid* mRNA that is injected into the oocyte localizes to the nearest region of anterolateral cortex. When treated with the nurse cell cytoplasm prior to the injection, it properly localizes only at the anterior (Cha et al., 2001). A nurse cell specific factor could bind to mRNA-protein complexes rendering it competent for anterior anchoring (Trovisco et al., 2016). A model that includes anchoring specifically at the anterior can explain why *bicoid* is not found at the lateral cortex. This model also predicts that the microtubule cytoskeleton does not need to be polarized for correct *bicoid* localization. Indeed, *bicoid* localizes correctly in the *shot* mutants, in which the microtubule cytoskeleton is not polarized (Trovisco et al., 2016). However, defects in *bicoid* mRNA localization occur in other mutants in which cytoskeleton polarization is compromised, such as *par-1*, *baz* and *grk* (González-Reyes et al., 1995; Benton et al., 2002; Doerflinger et al., 2010).

The mechanism of *oskar* mRNA anchoring is far better understood. First, cortical localization of *oskar* is reduced upon F-actin fragmentation (Cha et al., 2002) and depends on the actin-based motor, myosin-V (*didum* in *Drosophila*) (Krauss et al., 2009). This suggested that myosin-V could be part of an anchoring machinery. However, since both myosin-V and actin are uniformly distributed throughout the oocyte cortex, it was unclear how they can anchor *oskar* mRNA specifically at the posterior. To elucidate the mechanism of *oskar* mRNA transport and anchoring, Lu et al. (2020) analysed the localization of Staufen in different kinesin-1 and myosin-V mutants. According to the model proposed by Lu et al. (2020) (Figure 6, bottom), *oskar* mRNA is transported by kinesin-1 towards the plus–ends of microtubules, followed by anchoring at the posterior by myosin-V. A stochastic competition between kinesin dependent removal of *oskar* mRNA from the cortex along microtubules and myosin-V anchoring leads to differential steady-states along the oocyte cortex. While the density of microtubules is high at the anterolateral cortex, kinesin removal wins in that region. At the posterior, where nucleation of microtubules is suppressed, anchoring by myosin-V is predominant (Lu et al., 2020). This model explains why posterior localization of *oskar* can be achieved by myosin dependent anchoring, although myosin localization is not polarized. Furthermore, it explains why *oskar* localization critically depends on the polarization of the microtubule cytoskeleton.

## Future Perspectives and Focus Points

In *Drosophila*, the development from stem cell to mature oocyte is critically determined by a series of spatial decision-making processes. These processes go back to canonical cell polarity events governed by the Par protein network. Despite recent advances in our understanding of cell polarity and the downstream processes leading to cell selection and differentiation, intracellular reorganisation of the oocyte, and body axes formation, several major gaps of knowledge persist. In our view the most critical open questions relate to the interplay between follicle cells and the oocyte. Unlike in other species, for example in *C. elegans*, where polarization of the zygote and first body axis determination occurs cell autonomously, an intimate mechanistic relationship between soma and germline has been retained in *Drosophila*. Most importantly, the identification of the follicle cell signal that determines the second polarity event and body axis formation is long overdue, but all the past studies so far suggest that this would require analyses beyond genetic screens and knockout studies. Biophysical approaches that test the cell-cell interface between oocyte and posterior follicle cells could give new insight.
